# Global Modeled Sinking Characteristics of Biofouled Microplastic

**DOI:** 10.1029/2020JC017098

**Published:** 2021-04-22

**Authors:** Delphine Lobelle, Merel Kooi, Albert A. Koelmans, Charlotte Laufkötter, Cleo E. Jongedijk, Christian Kehl, Erik van Sebille

**Affiliations:** ^1^ Institute for Marine and Atmospheric Research Utrecht University Utrecht Netherlands; ^2^ Aquatic Ecology and Water Quality Management Group Department of Environmental Sciences Wageningen University Wageningen Netherlands; ^3^ Climate and Environmental Physics Physics Institute University of Bern Bern Switzerland; ^4^ Oeschger Centre for Climate Change Research University of Bern Bern Switzerland; ^5^ Department of Civil and Environmental Engineering Imperial College London London UK; ^6^ Centre for Complex Systems Studies Utrecht University Utrecht Netherlands

**Keywords:** Biofouling, Lagrangian, microplastic, modeling

## Abstract

Microplastic debris ending up at the sea surface has become a known major environmental issue. However, how microplastic particles move and when they sink in the ocean remains largely unknown. Here, we model microplastic subject to biofouling (algal growth on a substrate) to estimate sinking timescales and the time to reach the depth where particles stop sinking. We combine NEMO‐MEDUSA 2.0 output, that represents hydrodynamic and biological properties of seawater, with a particle‐tracking framework. Different sizes and densities of particles (for different types of plastic) are simulated, showing that the global distribution of sinking timescales is largely size‐dependent as opposed to density‐dependent. The smallest particles we simulate (0.1 μm) start sinking almost immediately around the globe and their trajectories take the longest time to reach their first sinking depth (relative to larger particles). In oligotrophic subtropical gyres with low algal concentrations, particles between 1 and 0.01 mm do not sink within the simulation time of 90 days. This suggests that in addition to the comparatively well‐known physical processes, biological processes might also contribute to the accumulation of floating plastic (of 1–0.01 mm) in subtropical gyres. Particles of 1 μm in the gyres start sinking largely due to vertical advection, whereas in the equatorial Pacific they are more dependent on biofouling. The qualitative impacts of seasonality on sinking timescales are small, however, localized sooner sinking due to spring algal blooms is seen. This study maps processes that affect the sinking of virtual microplastic globally, which could ultimately impact the ocean plastic budget.

## Key Points

- Sinking timescales of virtual particles subject to biofouling are more dependent on the initial size of particles than initial density
- Low algal presence in subtropical gyres and minimal biofouling can contribute to particles between 1 and 0.01 mm remaining at the surface
- Modeled biofouled particles of 0.1 microns start sinking almost immediately and show a global median sinking timescale of one day

## 1 Introduction

Estimates suggest that one hundred times more plastic enters the ocean annually than is found at the sea surface (Van Sebille et al., 2015). It is still unknown where the plastic goes and how much resides in the other ocean reservoirs, including the sea floor (Courtene‐Jones et al., 2020), the water column (Choy et al., 2019; Koelmans et al., 2017; Ye & Andrady, 1991), the shorelines (Lebreton et al., 2019) and marine biota (F. Galgani et al., 2015; Lusher et al., 2017). By improving our understanding of the ocean plastic budget, stakeholders including policy‐makers, conservationists, and scientists can focus their attention on the reservoir with the highest concentrations. The impact of plastic pollution on aquatic species as well as human health (e.g., through consumption of seafood (Smith et al., 2018) and sea salt (Kim et al., 2018)) can then also be further investigated.

Many recent studies on the transport of plastic in the ocean have focused on what occurs at the surface (Van Sebille et al., 2020). The three‐dimensional pathway of plastic, however, is still rather poorly understood (Mountford & Morales Maqueda, 2019). Around half of the mass of plastic produced worldwide is positively buoyant and therefore should float upon entry in the ocean (Andrady, 2011; Geyer et al., 2017). Some studies have shown, however, that plastic types with initial densities lower than seawater have been found in the water column and on the ocean floor (Courtene‐Jones et al., 2020; Hidalgo‐Ruz et al., 2012; Schwarz et al., 2019). When the density of a particle exceeds the density of its surrounding seawater, it begins to sink. A few processes could induce such sinking of microplastic by increasing its density (Van Sebille et al., 2020), including entrainment in marine snow (Michels et al., 2018; Porter et al., 2018; Zhao et al., 2018), fecal pellet sinking (Cole et al., 2016; Kvale et al., 2020), migration after ingestion by marine life (Botterell et al., 2019; Lusher et al., 2017), suspension in inorganic particles or metals (e.g., ambient toxins; Richard et al. (2019)) and algal growth or biofouling (Amaral‐Zettler et al., 2020; Kooi et al., 2017; Rummel et al., 2017). Here, we focus on the latter process since biofouling is recognized as one of the main potential sinks of surface microplastics and requires improved modeled estimates (Egger, Sulu‐Gambari, et al., 2020; Nguyen et al., 2020; Van Melkebeke et al., 2020).

Biofouling is the attachment and accumulation of biological organisms on the surface of a submerged object (Long et al., 2015; Ye & Andrady, 1991). The presence of plastic provides a new substrate for microbial communities to thrive (also known as the “plastisphere”; Wright et al. (2020); Zettler et al. (2013)). This can have a negative impact on other species in the euphotic zone via (1) reduced light penetration, (2) impaired oxygen and carbon dioxide production and (3) a thickened barrier for gaseous exchanges at the air‐sea interface. Upon sinking, the plastic induces a downward flux of organic carbon and hence can also affect the ocean’s biological and carbon pump (L. Galgani & Loiselle, 2020).

In this study, we estimate the sinking characteristics of particles on a global scale and therefore focus on a numerical modeling approach, since microplastic biofouling observations are still sparse and regional, as well as logistically hard to execute. The following are examples of locations where observational studies have been conducted; Biscayne Bay (Ye & Andrady, 1991), the Bay of Bengal (Artham et al., 2009), Cape Town (Fazey & Ryan, 2016), Germany (Kaiser et al., 2017) and Monterey Bay (Choy et al., 2019). Fazey and Ryan (2016) show that 17 days are required for 50% of low‐density polyethylene sheets of 5 × 5 × 0.1 mm^3^ to sink due to biofouling, and their smaller samples lose buoyancy much faster than their larger ones (49 days for 50 × 50 × 0.1 mm^3^).

Kooi et al. (2017) propose a model (hereafter referred to as the Kooi model) to estimate the sinking timescale of biofouled microplastic particles depending on their size, density, biofilm growth, and fixed depth profiles for water density, light, salinity, temperature, and viscosity. Their results suggest that under idealized hydrodynamic conditions, due to a trade‐off between the collision frequency with algae and surface‐to‐volume ratio, the smallest particles (0.1 μm) start sinking immediately and the larger particles (0.1–10 mm) take around 30 days to start sinking. Their study explores the vertical transport of microplastic at a fixed latitude and longitude, hence the motivation behind our study to investigate the three‐dimensional transport while using more realistic hydrodynamic and biological conditions that can vary temporally and spatially.

The aim of our work is to generate a global map of the sinking characteristics of initially buoyant virtual particles subject to biofouling. To improve understanding of the fate of floating microplastic in the ocean, the sinking characteristics we explore are (1) the residence time at the surface before sinking (referred to as the sinking timescale) and (2) the time it takes to sink to a depth when its vertical velocity is no longer oriented downwards (referred to as its first sinking depth). All processes that could affect transport below the surface after the virtual particle reaches its first sinking depth are therefore beyond the scope of this current work. Furthermore, three‐dimensional advection (horizontal and vertical) is included in this study, however, any effects of turbulence on the sinking characteristics are not included.

We explore the sinking response of the virtual particles to the following parameters: particle size, particle density, three‐dimensional advection, global seawater properties (temperature, salinity and density) and biological properties (algal concentrations and growth), the mixed layer depth (MLD) and seasonality. The initial particle size and density are defined at the start of each run and the other parameters vary dynamically in time and space, affecting the particles’ size and density due to the growth of the biofilm. Regarding the particles' size, “microplastic” is now commonly defined among the scientific community as <5 mm, and here we define it as spherical particles that have a radius between 1 mm and 0.1 μm, which almost covers the same size range used in Kooi et al. (2017). We also use three initially buoyant densities (representing commonly used polymer types). We have therefore further developed the one‐dimensional (vertical) Kooi model, and present a Lagrangian analysis of the global three‐dimensional sinking characteristics of biofouled microplastic. Implications of this work include bringing us closer to understanding the potential sinking fate of “missing” floating plastic, due to size‐selective sinking of particles as a result of biofouling and large‐scale advection, that can vary in different locations of the global ocean.

## 2 Methods

### 2.1 Lagrangian Model Set‐Up

The three‐dimensional transport of microplastic is simulated using the Lagrangian particle‐tracking framework, OceanParcels Version 2.1.6 (Delandmeter & van Sebille, 2019). We use the NEMO‐MEDUSA‐2.0 ORCA0083‐N06 output (Yool et al., 2013), hereafter MEDUSA, for the hydrodynamic data and biogeochemical data, which has a 1/12° global horizontal resolution and 75 depth levels. The data include the three‐dimensional ocean velocity fields, temperature, salinity, phytoplankton concentrations and primary productivity (the latter two are converted to algal concentrations and biofilm growth, explained in Section 2.2). We use five‐day averages from 2000 to 2009 (available from http://opendap4gws.jasmin.ac.uk/thredds/nemo/root/catalog.html). It is therefore important to note that we are investigating the effects of large‐scale advective features (such as upwelling, downwelling, convergence, and divergence). Previous work has shown that Lagrangian experiments using temporal means of up to nine days do not exhibit any significant degradation in these large‐scale flow characteristics (Qin et al., 2014).

Particles are released on a global 2° × 2° grid (9,620 particles) from 70°S to 80°N. The full latitudinal range is not used due to interpolation challenges when using the tripolar MEDUSA grid. The particles are released at 0.6 m (the surface depth in MEDUSA) and we simulate the particle trajectories for 90 days forward in time. The Lagrangian framework provides spatial and temporal interpolation of the fields following the C‐grid interpolation scheme (Delandmeter & van Sebille, 2019). We use the three‐dimensional fourth‐order Runge‐Kutta method with an integration time step of 30 s and the three‐dimensional position of each particle is stored every 12 h (180 time steps per simulation). Five sizes are chosen for the microplastic with a radius from 1000 to 0.1 μm (decreasing by one order of magnitude). Three different densities representing different initially buoyant plastic types are used; expanded polystyrene (EPS = 30 kgm^−3^), polypropylene (PP = 840 kgm^−3^) and low‐density polyethylene (LDPE = 920 kgm^−3^). We assume that all virtual microplastic particles are spherical and initially pristine, as in the Kooi model. It should be noted that our results are subject to the parametrizations and design of the model and that for example, the assumption of microplastic being present at the sea surface as clean and spherical must be taken into consideration when interpreting the results. To optimize efficiency (and reduce intensive computing time), we choose to run simulations for the “most typical year” from the 10 years mentioned above; 2004 (see Section S1 for a description on how we determine that year).

Two regional simulations are additionally generated in order to evaluate the effects of removing the biofouling process and removing advection on the sinking of the particles, where 25 particles are released on a 10° x 10° grid (with a 2° x 2° horizontal resolution). One region is in the algal‐rich zone of the equatorial Pacific (EqPac), from 4°S‐4°N and 140°W–148°W. The other region is in the algal‐scarce North Pacific subtropical gyre (NPSG), from 28°N‐36°N and 135°W–143°W. This latter region has been chosen since it is shown to have the highest concentrations of marine debris in the NPSG from Manta trawl samples (Egger, Nijhof, et al., 2020). We also choose to run this second simulation set‐up in the NPSG for 1000 days, from January 1, 2004, in order to test whether a longer simulation time allows for virtual microplastic particles to leave the accumulation zone. Lastly, these 1000 days simulations are repeated with horizontal advection only and vertical advection only, to isolate the effects of the different components of the three‐dimensional velocity fields.

### 2.2 Kooi Model

The equations governing the biofouling and sinking of microplastic in this study are based on the Kooi model (see Kooi et al. (2017) for the detailed method). Our code to generate the results can be found here: https://doi.org/10.5281/zenodo.4543145. The sinking velocity, *V*_*s*_ (ms^−1^), is dependent on the density difference between the combined plastic plus biofilm and its surrounding seawater, as well as the buoyancy of the virtual microplastic (i.e., the particle’s size and density):

(1) $$V_{s}\left(x,y,z,t\right)=-{\left(\frac{\rho_{tot}-\rho_{sw}}{\rho_{sw}}g\omega_{\;*\;}\upsilon_{sw}\right)}^{1/3}$$

where *ρ*_tot_ is the total density of the particle plus attached algae (kgm^−3^), *ρ*_sw_ is the ambient seawater density (kgm^−3^), which is a function of the three spatial directions and time (*x*, *y*, *z* and *t*), *g* is the gravitational acceleration (ms^−2^), ω_*_ is the dimensionless settling velocity and *υ*_sw_ is the kinematic viscosity of the seawater (m^2^s^−1^). The total density, *ρ*_tot_ (Equation S11), is calculated from the radius (hereafter referred to as ‘size’) of the particle (m), the volume of an algal cell (López‐Sandoval et al., 2014), the biofilm thickness (m), and the biofilm and plastic density (kgm^−3^) (Equation S6–S10). The seawater density, *ρ*_sw_, is calculated as a function of conservative temperature and absolute salinity (see Roquet et al. (2015) and McDougall et al. (2003) for in‐depth explanations of TEOS‐10 standard equation of state). As mentioned above, we use temperature and salinity profiles from the MEDUSA model output that vary in time and three‐dimensional space. Using a more realistic representation of the global hydrodynamic estimates allows us to further develop the idealized one dimensional Kooi model that have profiles that only vary with depth. The dimensionless settling velocity, ω_*_, is a function of the dimensionless particle diameter (Equation S18–S21) and we keep *υ*_sw_ profiles as defined in the Kooi model (Equation S1–S5; following Sharqawy et al. (2010)). Variations in kinematic viscosity are shown to be negligible for temperatures found in the ocean (Chen et al. (1973); see Section S2).

The attached algal growth, d*A*/d*t* (no. m^−2^s^−1^), is dynamically estimated as:

(2) $$\frac{dA}{dt}=\frac{A_{A}\beta_{A}}{\theta_{pl}}+\mu_{A}A-m_{A}A-Q_{10}^{\left(T-20\right)/10}R_{20}A$$

where *A*_*A*_ is the ambient algal concentration (no. m^−3^), *β*_*A*_ is the encounter kernel rate (m^3^s^−1^), *θ*_*pl*_ is the surface area of the spherical plastic (m^2^), *μ*_*A*_ is the growth of the attached algae (s^−1^), *m*_*A*_ is the grazing or mortality rate (s^−1^) and the final term accounts for respiration. The ambient algal concentration, *A*_*A*_, is estimated by converting the MEDUSA output of diatom plus non‐diatom phytoplankton concentrations (mmol Nm^−3^) into number of algal cells per unit volume (no. m^−3^). This is calculated by first multiplying the total phytoplankton concentrations by the atomic weight of 1 mol of nitrogen (i.e., 14.007 g, to obtain mg Nm^−3^). Then, for the nitrogen to algal cell conversion, the median value in the literature (356.04 × 10^9^) is used, reported by Menden‐Deuer and Lessard (2000). The median value is also used in the Kooi model, however they use a carbon to cell conversion method since they estimate the theoretical depth‐profile of chlorophyll‐a concentration (and then compute the chl‐a/carbon and carbon/algal cell conversions). The encounter rate, *β*_*A*_, represents the collision of the particle with water, and if the water contains algae (from *A*_*A*_), we assume that the algae attaches to the particle. This *β*_*A*_ term is calculated as the sum of collision frequencies from Brownian motion, differential settling and advective shear (Equation S15–S17; which are dependent on diffusivity equations from Jackson (1990)). MEDUSA’s total primary productivity (mmol Nm^−3^ d^−1^) is converted to attached algal growth, *μ*_*A*_, by doing the same conversions as above for *A*_*A*_ (multiplying by 14.007 and dividing by 356.04 × 10^9^), however, the final step includes dividing by this algal cell concentration in order to isolate the growth factor (in d^−1^, and then converting to s^−1^). While in Kooi et al. (2017) only temperature and light limit algal growth, the MEDUSA output also considers nutrient limitation by nitrate, silicon, and iron (Yool et al., 2013). Another difference between the Kooi model and this study is that the hourly Kooi growth rate reflects a daily cycle of light intensity, while MEDUSA output is only available as five‐day averages, masking the daily cycle. However, we expect the impact on the average daily effects to be negligible for sinking timescales longer than 1 day. Since the Kooi et al. (2017) results are based on a light/dark ratio that is equal (12 h each), our average global MEDUSA‐based sinking timescales should be similar to their study (which we show is the case below). The mortality and respiration terms have been modeled identically to the Kooi model using constant rates of *m*_*A*_ = 0.39 d^−1^ and *R*_20_ *A* = 0.1 d^−1^ with a *Q*_10_ coefficient = 2, which represents how much the respiration rate increases by every 10°C increase in temperature; where *T* is temperature (°C) from MEDUSA. Since the aim of our study is to incrementally increase the complexity of the Kooi model with three‐dimensional data available from MEDUSA, processes such as grazing, mortality, and respiration are still parametrized following the Kooi model.

It should be mentioned at this stage that the Kooi equations produce unrealistic results for the sinking velocities of 10 mm size particles (which reach a *V*_*s*_ value of 20 ms^−1^). Hence, we only focus on particles of a 1 mm particle radius (that have a maximum *V*_*s*_ of 0.2 ms^−1^) and smaller. Furthermore, within the context of the Kooi model, any effects of turbulence are not included in this study. This has been decided due to the fact that a wind‐driven mixing term proposed by Kukulka et al. (2012) used in previous studies (e.g., Wichmann et al. (2019)) does not hold for negatively buoyant particles, which is the case for biofouled particles that sink in our study. Parametrizations of mixing (such as wind‐driven mixing or sub‐grid processes) must therefore be further developed in future work and have been left out of the current study. Furthermore, the advantage of this set‐up for our process study is to separate the effects of advection from wind‐driven mixing. We would expect that if one were to include vertical mixing to our study, particles with a smaller sinking velocity than that of the mixing term would exhibit shorter sinking timescales (for example, the smallest particles) and could sink deeper.

Our results are represented using two quantities. The first is the sinking timescale, *T*_*s*_, defined as the number of days until the vertical velocity (*V*_*s*_ from Equation 1) plus the vertical advection from MEDUSA is downward, within the simulation time of 90 days. Since the particles are all initially positively buoyant, we force any rising particles to remain at 0.6 m (the ‘surface depth’ in MEDUSA), with a vertical velocity of 0 m s^−1^. This is to avoid algal attachment due to oscillations between 0 and 0.6 m (see Equation S17) that are meaningless since we treat 0.6 m as our surface. Simulations for the four seasons are first run separately, between December 2003 and November 2004 inclusive (released on December 1st, 2003 for DJF, December‐January‐February; and so on for MAM, March‐April‐May, JJA, June‐July‐August and SON, September‐October‐November). The four *T*_*s*_ maps produced have been averaged at each initial release location (for only those simulations where particles do sink within the 90 days) in order to produce one global map of *T*_*s*_ estimations. These *T*_*s*_ maps are therefore plotted where the particles are first released (as opposed to the location where the particles first sink, since it would be harder to interpret particles that overlap spatially). The first sinking depth, *Z*_*s*_, for each individual season is defined as the depth each particle reaches until its vertical velocity is no longer downwards. We do this to highlight the initial effects of biofouling on the sinking of particles that are originally found at the surface, rather than the final position of the particles. If a submerged particle remains at a certain depth, or ascends, that part of the trajectory is not included in this study. Furthermore, if a particle continuously sinks throughout the 90 days and its vertical velocity remains downwards, its depth at 90 days is recorded as *Z*_*s*_. We therefore capture each particle’s trajectory as a function of depth and time until *Z*_*s*_, its first sinking depth.

## 3 Results and Discussion

### 3.1 Sinking Timescale

**Figure 1:**
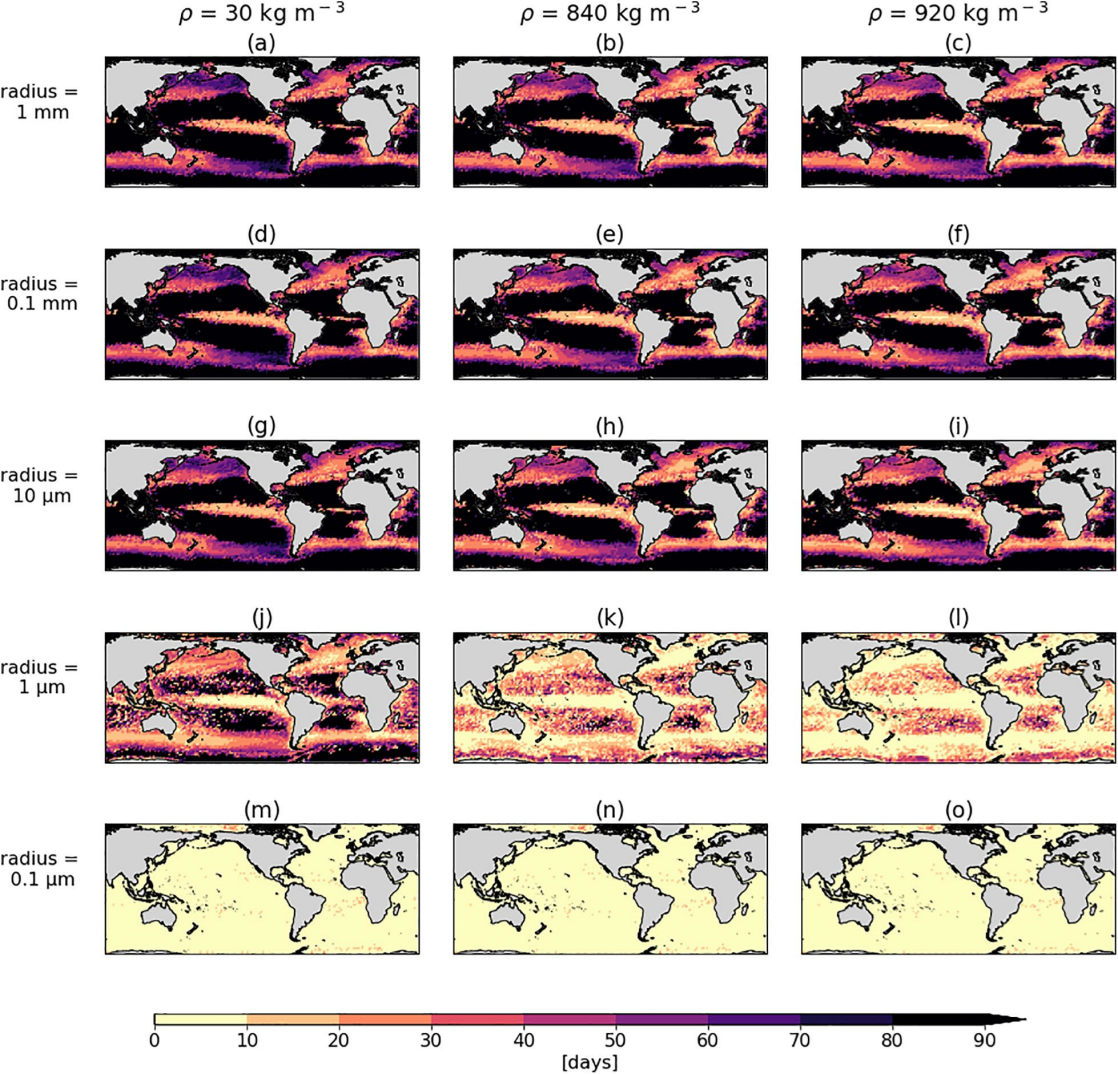
The sinking timescale, *T*_*s*_ (days) over simulations of 90 days for virtual microplastic particles of different sizes and densities. The left column shows results of *T*_*s*_ for EPS (30 kgm^−3^), the middle column for PP (840 km^−3^) and the right column for LDPE (920 kgm^−3^). The rows represent the sizes of particles, with a radius of: (a)–(c) 1 mm, (d)–(f) 0.1 mm, (g)–(i) 10 μm (j)–(l) 1 μm and (m)–(o) 0.1 μm. The *T*_*s*_ is plotted at each 2° x 2° grid initial release location (at 0.6 m depth) and the black colors represent no sinking within 90 days. The average *T*_*s*_ from the four seasons in 2004 is shown. EPS, expanded polystyrene; LDPE, low‐density polyethylene; PP, polypropylene.

The globally mapped sinking timescale (*T*_*s*_) as a result of biofouling and three‐dimensional advection is estimated for different virtual microplastic sizes and densities (Figure 1). A particle sinks as soon as its density exceeds that of its surrounding seawater density and vertical advection is downwards. As explained in Kooi et al. (2017), the sinking timescale is a trade‐off between the particle’s size and the surface‐to‐volume ratio. The larger particles are more likely to encounter ambient algal cells, hence the collision rate is higher and the density can increase sooner. On the other hand, the smaller the object, the greater the relative surface area, meaning that the attachment of a very small number of ambient algae can cause it to sink. This concept can be visualized by the results for the smallest particles simulated here (0.1 μm in Figures 1m, 1n, and 1o) where particles start sinking almost immediately globally (median global *T*_*s*_ = 1 day for all three densities; 30, 840 and 920 kgm^‐3^). The second‐smallest particles (1 μm in Figures 1j, 1k, and 1l) take slightly longer in some regions than the smallest particles, and then the three largest sizes (1, 0.1 and 0.01 mm in Figures 1a–1i) produce almost identical global *T*_*s*_ maps. For these three largest sized particles, the median global *T*_*s*_ is 40–43 days for the density particle type, LDPE (Figures 1c, 1f and 1i), 35–38 days for PP (Figures 1b, 1e and 1h) and 33–36.5 days for the least dense, EPS (Figures 1a, 1d and 1g; although these differences are not statistically significant, with a standard deviation of ±18 days for all nine global maps). Our results mirror the one dimensional Kooi et al. (2017) results in that an asymptotically shaped relationship between the log of increasing particle sizes versus the sinking timescale is reached in almost all locations.

Another result to highlight is what occurs in the five subtropical gyres. For the larger sizes in this study (1 mm–10 μm in Figures 1a–1i) the subtropical gyres are distinguished from the other regions by the fact that the particles do not sink within the simulation time of 90 days (black patches). This behavior can be attributed to the subtropical gyres being largely oligotrophic zones (Morel et al., 2010), implying that there is low biological activity (lighter green patches in Figure 3, left column). This leads to low algal collision frequencies such that the density of the particles does not exceed that of its surrounding seawater. On the other hand, since less algal attachment is required to increase the density of smaller particles (1 μm in Figure 1j–1l and 0.1 μm in Figure 1m–1o), the small particles in the subtropical gyres do sink within the 90 days (albeit later than in the other oceanic regions for 1 μm particles). Even when extending this to a regional simulation of 1000 days, only 2 of the 25 particles sink for LDPE particles of 1 mm (Figure S3). This suggests that as a result of the biofouling process, larger microplastic particles can be more present at the surface than smaller particles in subtropical gyres. This follows the size‐selective removal theory proposed by Cózar et al. (2014) and further supported by Egger, Nijhof, et al. (2020). Furthermore, for all particle sizes, the equatorial regions show the shortest sinking timescales of <10 days, which coincides with regions of higher algal concentrations (darker green patches in Figure 3, left column; regardless of the season). These subtropical and equatorial results prompt us to run regional simulations to better understand *T*_*s*_ in regions of extreme high and low algal concentrations and with different large‐scale physical processes, such as upwelling and downwelling (see Section 3.4).

Lastly, the qualitative differences in *T*_*s*_ as a function of size are more distinguishable than the differences in *T*_*s*_ for different densities of microplastic. The most sensitive particle size to density differences is 1 μm; in the subtropical gyres the 1 μm particles of 30 and 840 kgm^−3^ take longer to sink (up to twice as long, if they sink at all within 90 days), relative to 920 kgm^−3^ particles (Figures 1j and 1k relative to 1l). The general mapped patterns of *T*_*s*_ for the three densities are otherwise very similar globally for the other size classes and hence our results demonstrate that the global modeled distribution of sinking timescales of microplastic is very similar for different types of initially buoyant plastic (including EPS, PP, LDPE). This is somewhat expected due to the fact that the sizes can range over many orders of magnitude (five in this case) and the densities do not even reach two orders of magnitude (due to the definition of buoyant plastic types being below the average density of seawater: 1025 kgm^−3^).

### 3.2 First Sinking Depth

**Figure 2:**
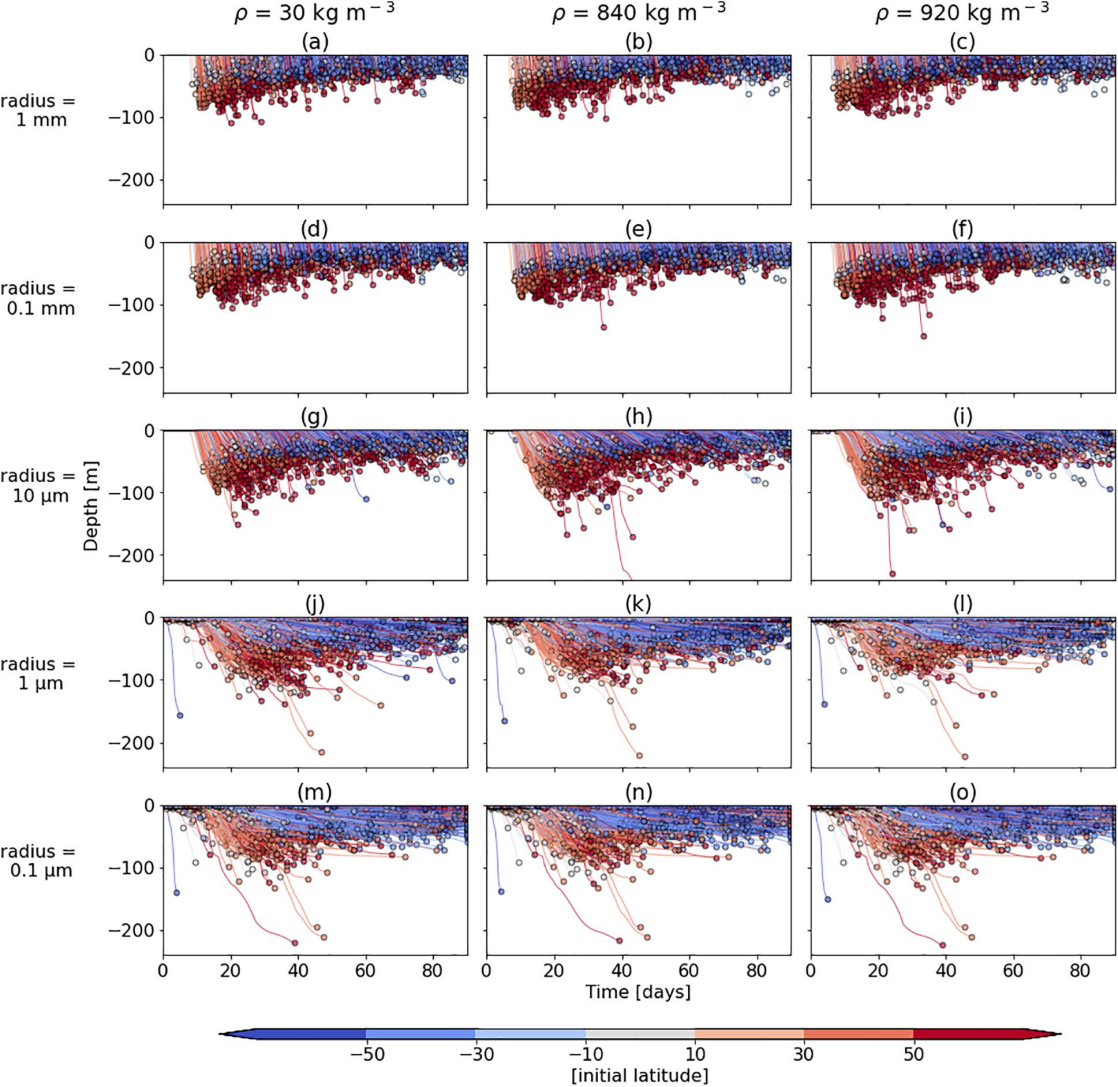
The first sinking depth, *Z*_*s*_ (m), over 90 days for the same initial particle densities and sizes as in Figure 1. The depths of each particle’s trajectory over time are shown until *Z*_*s*_ (the dots at the end of each line). The colors represent the latitude at which each particle is initially released, with red colors for the northern hemisphere and blue colors for the southern hemisphere. Here, only one simulation season is used (MAM) so as to not isolate the effect of each season on *Z*_*s*_ yet. All particles that do not sink within 90 days are not plotted.

For the *Z*_*s*_ results, we use one of the seasons as an example; boreal spring (March‐April‐May; MAM). The deeper *Z*_*s*_ (m) and sooner *Z*_*s*_ timescale (days) for the particles of all sizes released in the northern hemisphere (red trajectories in Figure 2) can be explained by the deeper mixed layer depth and larger algal blooms during the spring in the northern Atlantic. To clarify, the *Z*_*s*_ timescale includes both *T*_*s*_ (days) as well as the time it takes from leaving the surface to reaching *Z*_*s*_. The effect of seasonality on both *T*_*s*_ and *Z*_*s*_ is explored further in Section 3.3.

Although as stated in the previous section, *T*_*s*_ is almost identical globally for the three largest sizes, the time it takes to reach the first sinking depth, *Z*_*s*_, is not identical for those three sizes (1–0.01 mm in Figures 2a–2i). Rather, it gradually takes longer to reach *Z*_*s*_ with a decrease in particle size. For example, the largest‐sized virtual LDPE particles can sink from the surface to around 100 m within a few days (Figure 2c), whereas the smallest‐sized virtual particles can take 40 days to reach a similar depth (Figure 2o), and the latter start sinking almost immediately around the globe. Once again, as with the *T*_*s*_ results, the difference in *Z*_*s*_ for the different initial densities is very small, therefore all further simulations have used the density of LDPE; 920 kgm^−3^.

### 3.3 Effects of Biological and Physical Seasonality on Sinking Characteristics

**Figure 3:**
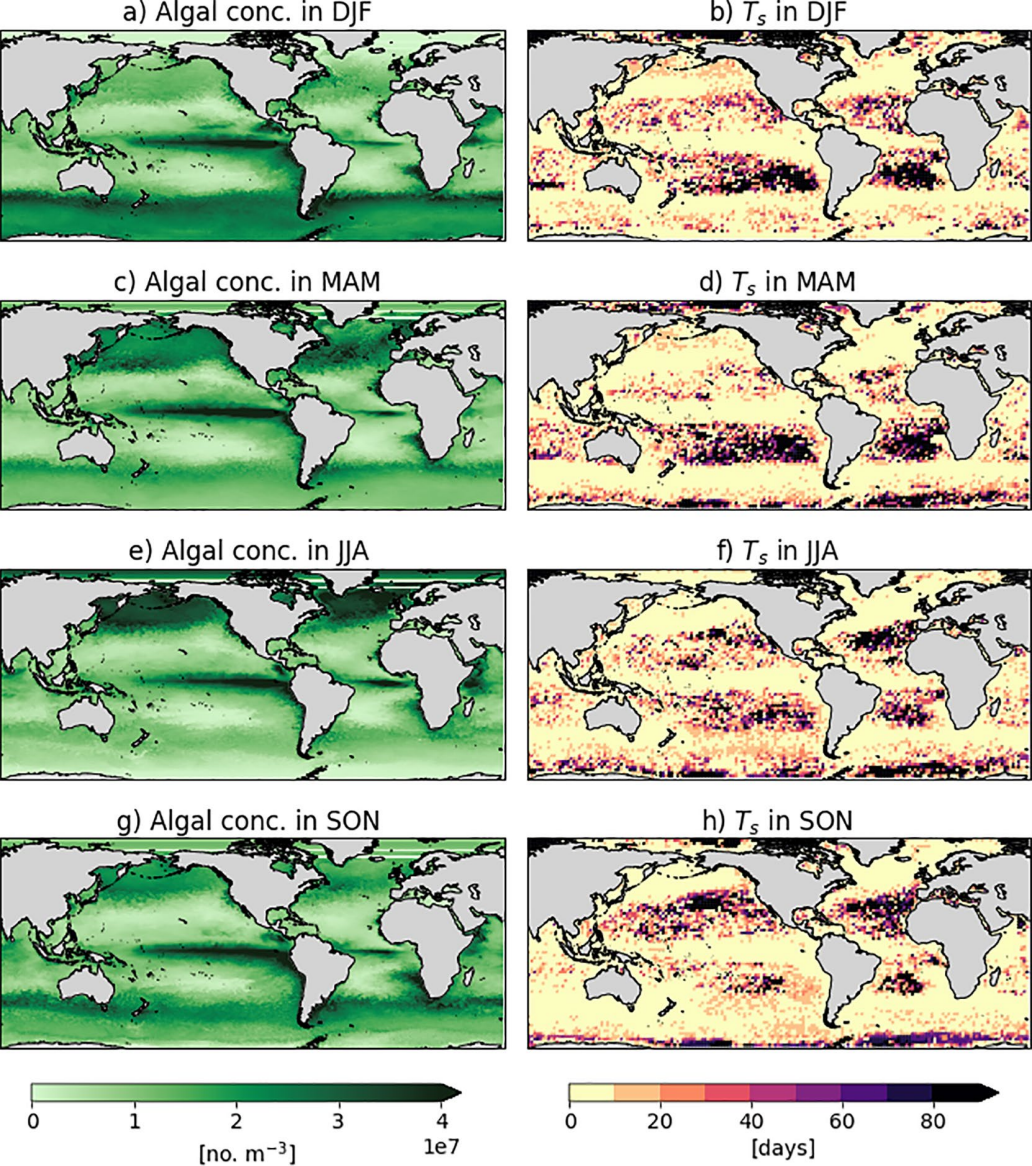
Surface algal concentrations (no. m^−3^) derived from the MEDUSA output (left column) and sinking timescale (*T*_*s*_ in days, right column) for LDPE particles of a 1 μm radius. Seasonal averages of algal concentration are displayed: DJF in (a), MAM in (c), JJA in (e) and SON in (g) for 2004 (the “typical year” used in this study). Note that the MEDUSA output consists of phytoplankton concentrations that have been converted to algal concentrations for the Kooi model equations; see Methods for further explanation. The results for *T*_*s*_ represent one simulation each, which is run for 90 days starting at the beginning of each season: DJF in (b), MAM in (d), JJA in (f) and SON in (h). These four *T*_*s*_ maps are averaged to generate Figure 1l, and the colorbar is as in Figure 1. DJF, December‐January‐February; MAM, March‐April‐May; JJA, June‐July‐August; SON, September‐October‐November; LDPE, low‐density polyethylene.

**Figure 4:**
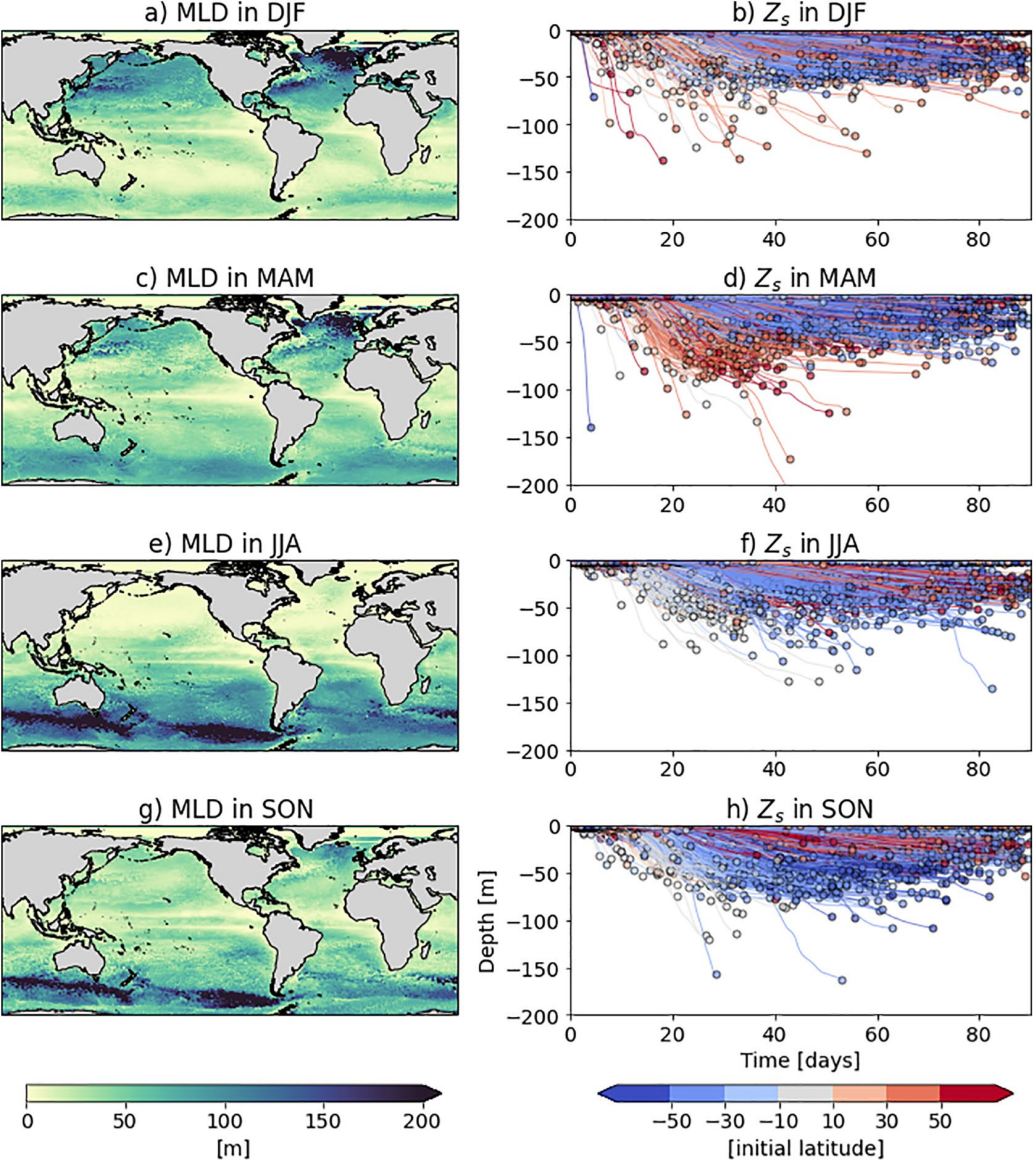
Mixed layer depth from the MEDUSA output (MLD in m; left column) and first sinking depth (*Z*_*s*_ in m, right column) for LDPE particles of a 1 μm radius. Seasonal MLD averages are displayed: DJF in (a), MAM in (c), JJA in (e) and SON in (g) for 2004 (the “typical year” used in this study). The results for *Z*_*s*_ represent one simulation each, which is run for 90 days starting at the beginning of each season (as in Figure 3). (d) Here is identical to Figure 2l, being for particles of a radius of 1 μm. The colorbar for the right column is as in Figure 2. DJF, December‐January‐February; MAM, March‐April‐May; JJA, June‐July‐August; SON, September‐October‐November; LDPE, low‐density polyethylene; MLD, mixed layer depth.

Sinking characteristics of particles can be influenced by biological and physical features, such as ambient algal concentrations and the mixed layer depth (Figure 3 and Figure 4). Since we release the virtual particles at 0.6 m, we show surface algal concentrations to illustrate the surrounding algae available for initial attachment and growth that influences when particles start sinking (i.e., *T*_*s*_). The MLD is shown since waters maintain generally homogeneous properties from the surface to the base of the mixed layer. With a deeper mixed layer, for example, a deeper constant seawater density profile from the sea surface could result in sinking particles having a deeper *Z*_*s*_ since sudden changes in surrounding density are not encountered (Figure 4).

In this study, we distinguish the subtropical gyres as the regions that maintain the lowest surface algal concentrations across all seasons (which are consistent with previous work defining the convergence zones; e.g., Figure 4d in Maximenko et al. (2012)). Using one initial particle size (1 μm) to isolate effects of seasonality on the sinking timescale (Figures 3b, 3d, 3f, and 3h), the regions with higher algal concentrations generally correspond to shorter *T*_*s*_ (equatorial regions, Southern Ocean and northern Atlantic and Pacific), while lower concentrations correspond to a longer *T*_*s*_ (subtropical gyres) across the seasons (this is also seen for other sizes, e.g. 0.1 mm in Figure S1). Focusing on one season, the spring is known to influence algal concentrations due to algal blooms in MAM in the northern hemisphere and SON in the southern hemisphere (Figures 3c and 3g, respectively). During boreal spring, the North Atlantic and North Pacific show the lowest *T*_*s*_ across all seasons, with some particles sinking within 10 days in the subtropical gyres, while the southern hemisphere subtropical gyres show *T*_*s*_ values of up to 90 days or more (Figure 3d). Similarly, during austral spring (SON) the southern hemisphere subtropical gyres show comparatively lower *T*_*s*_ values relative to the other seasons, reducing the patches with *T*_*s*_ > 90 days (Figure 3h).

Regarding the *Z*_*s*_ results as a function of seasonality (Figure 4), the spring months show consistency with *T*_*s*_ results. During MAM, 1 μm particles released in the northern hemisphere reach their *Z*_*s*_ sooner (Figure 4d), and during SON, particles released in the southern hemisphere reach their *Z*_*s*_ sooner (Figure 4h). This is also seen for all other sizes (for example, 0.1 mm particles in Figure S2). The maximum *Z*_*s*_ reached for particles released in the northern and southern hemisphere is also deeper for their respective spring months relative to the winter or summer months. This once again suggests that during spring, particles sink deeper with higher surface algae present (Figure 3c and 3g, respectively) and with deeper MLDs (Figure 4c and 4g, respectively).

Although *T*_*s*_ differences have been mentioned for different seasons, it is important to highlight that the results across the seasons are generally fairly similar. We can therefore further justify averaging the four seasons for each of the sizes in Figure 1. Furthermore, other physical and biological parameters from MEDUSA have also been analyzed, such as the euphotic layer depth and the total primary productivity (which is converted to algal growth for our model), however, they produce very minor global distribution differences across the four seasons (not shown), hence the focus on MLD and algal concentrations.

### 3.4 Regional Simulations Analyzing Advection and Biofouling

**Figure 5:**
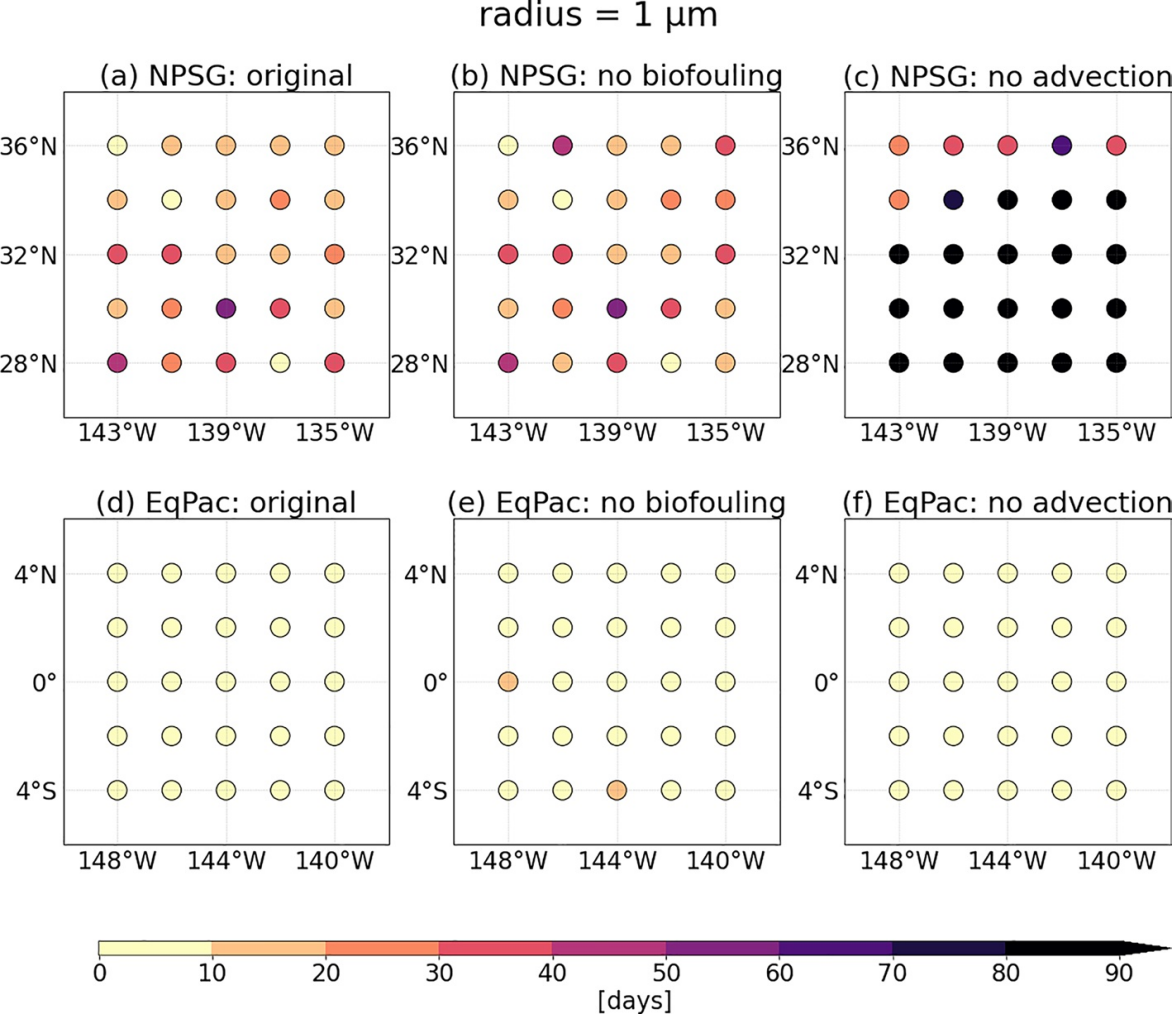
The sinking timescale (*T*_*s*_ in days) for two regional analyses: the North Pacific subtropical gyre (NPSG) in (a), (b) and (c) and equatorial Pacific (EqPac) in (d), (e) and (f) for LDPE particles of a 1 μm radius. The left column is the original set‐up of the simulations, as in Figure 1 (with advection and biofouling), the middle column shows the effects of removing biofouling, and the right column shows the effects of removing three‐dimensional advection. These are the results from averaging the four simulations for each season in 2004 that are run for 90 days. The colorbar for the sinking timescale is as in Figure 1. LDPE, low‐density polyethylene.

We now isolate the effects of advection and biofouling on the sinking characteristics by comparing regional results with and without these processes. Since the subtropical gyres are a recurring region of interest in this study, we choose to compare *T*_*s*_ in the oligotrophic North Pacific subtropical gyre (NPSG) to *T*_*s*_ in the algal‐rich equatorial Pacific (EqPac). Furthermore, the particle size that deserves a more in‐depth analysis is the 1 μm, since particles sink in the NPSG within 90 days (Figure 5a), contrary to particles larger than 1 μm (Figure 1).

**Figure 6:**
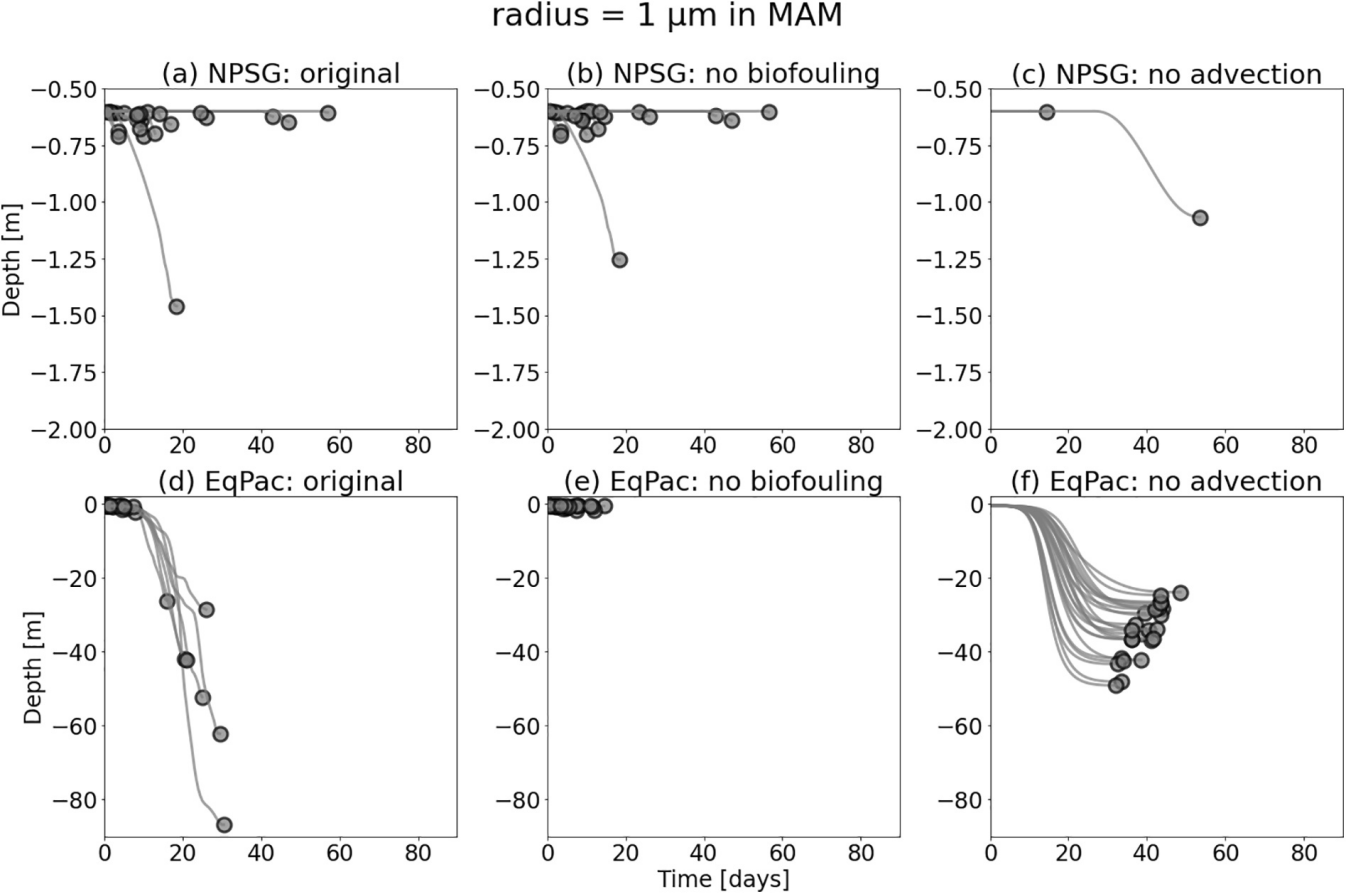
The first sinking depth, *Z*_*s*_ (m), over 90 days for the same regional analyses as in Figure 5. The depths of each particle’s trajectory over time are shown until *Z*_*s*_ (the dots at the end of each line). One season is used for these simulations; MAM. Note the different depth scales in the first and second rows. MAM, March‐April‐May.

In the NPSG simulation without biofouling while keeping advection (Figure 5b), *T*_*s*_ slightly increases relative to simulations with both biofouling and advection (Figure 5a). The simulation lacking advection while keeping biofouling (Figure 5c) results in almost all of the 25 particles staying at the surface throughout the 90 days. This suggests that three‐dimensional advection is the process with the largest impact on *T*_*s*_ for 1 μm in the NPSG. When repeating these simulations for 1000 days, and running one simulation with horizontal advection only and the other with vertical advection only, we see that vertical advection specifically plays a key role in transporting 1 μm particles below the surface in this downwelling zone (Figure S5d vs. Figure S4d). Since algal concentrations are low throughout all seasons (Figure 3) in the NPSG, the fact that removing biofouling has a lower impact on *T*_*s*_ is somewhat expected. The *Z*_*s*_ results further confirm this; those 1 μm particles that do sink within the 90 days of the MAM simulation, have a very shallow *Z*_*s*_ (<2 m in Figure 6a), and removing biofouling has a very minor impact on this first sinking depth (Figure 6b). If particles of all sizes are fixed to the northern edge of the NPSG (without horizontal advection), the particles can be exposed to more algae than those that are advected toward the center of the gyre, and hence can sink after > 700 days (Figure S5).

In the EqPac, although the presence of algae is consistently high throughout the year (Figure 3), *T*_*s*_ is almost not affected by the removal of biofouling or advection for 1 μm particles (*T*_*s*_ < 10 days in Figures 5d–5f). When analyzing the impacts of these processes on *Z*_*s*_ (for MAM), however, if local advection in this upwelling region is isolated (and biofouling is removed), very shallow first sinking depths are produced (*Z*_*s*_ < 2 m; Figure 6e) whereas *Z*_*s*_ can otherwise reach 20‐50 m when advection (mainly upwelling here) is removed (Figure 6f).

## 4 Conclusion

In this study, we simulate the effects of biofouling on the global sinking timescales of initially floating virtual microplastic (with a radius of 1 mm to 0.1 μm). Sinking of buoyant particles in the marine environment depends on the density difference between the particle and the surrounding seawater, as well as downward vertical advection for smaller particles. Therefore, particle properties combined with the global temporal and spatial variability of physical and biological properties can result in different sinking timescales in different regions of the ocean. The factors that have been analyzed here include the initial radius and density of microplastic, the seasonality of algal concentrations and mixed layer depth and the presence of three‐dimensional advection. We have therefore expanded on the Kooi et al. (2017) study that models the theoretical effects of biofouled particles in the vertical dimension and now map three‐dimensional, global sinking characteristics.

Our results suggest that the sinking of virtual particles subject to biofouling is largely dependent on the ambient algal concentrations as well as the initial size of the particle (rather than the initial density). The 1 μm particles seem to be the most sensitive to different particles’ initial densities, where the sinking timescale in subtropical gyres can half for the densest particles (920 kgm^−3^) relative to the least dense (30 kgm^−3^). The smallest virtual particle size analyzed here (0.1 μm) shows an almost immediate sinking timescale in the global ocean (with a median of less than a day). The smaller the particles, however, the longer they take to sink to their first sinking depth. Spring blooms (in both hemispheres) can lead to localized regions with higher surface algal concentrations and in turn shorter particle sinking timescales. Despite this, the overall qualitative distribution of *T*_*s*_ is quite similar across seasons. We also hypothesize that the combination of high algal concentrations and deeper mixed layers can reduce the time particles take to reach *Z*_*s*_.

Although the median global *T*_*s*_ matches the one dimensional results from Kooi et al. (2017) (1 day for 0.1 μm to 30–40 days for 10 μm to 1 mm), the novel aspect of our study is the global map we provide of *T*_*s*_. It allows us to explore different regions with different biological and physical features. The most prominent result in the global *T*_*s*_ maps of particles between 10 μm and 1 mm is the lack of sinking after 90 days in low‐productive (oligotrophic) regions where particles have a low encounter rate with algae. Even for a simulation of 1000 days in the oligotrophic North Pacific subtropical gyre, only 2 out of 25 particles of a 1 mm size manage to sink. A more detailed analysis into the smaller particles suggests that *T*_*s*_ and *Z*_*s*_ of 1 μm particles are more dependent on advection than biofouling in subtropical gyres. In other regions with higher algal concentrations (e.g., the equatorial Pacific) *T*_*s*_ is shorter than in the NPSG. Upwelling can keep particles from having a deep *Z*_*s*_ in the EqPac, and therefore a deeper *Z*_*s*_ occurs when biofouling is dominant. Our results hint to the fact that particles between 10 μm and 1 mm might be accumulating in the five subtropical gyres due to both physical reasons as well as biological properties (i.e., the lack of algae to make them sink) as also implied by Egger, Nijhof, et al. (2020). This supports the size‐selective removal theory of surface particles as proposed by Cózar et al. (2014) and seen in observations in Fazey and Ryan (2016), where smaller particles are found at a lower concentration at the sea surface than expected since they sink sooner.

In this study, we focus on implementing the one dimensional Kooi model in a more realistic biogeochemical and hydrodynamic setting by using temporally and spatially varying output data from MEDUSA. Both the choice of particle transport equations (e.g., drag parameterizations depending on shape, size, surface roughness, etc.) and environmental parameters (e.g., mixing strength) put constraints on the particle size range it is valid for (e.g., Monroy et al. (2017); De la Fuente et al. (2020)). Further work is required to determine the effects of these parameters as well as replacing the “pristine, spherical” particles with different shapes or fragmentation properties (e.g., DiBenedetto et al. (2019)) once they are further parametrized in the literature. Although our aim is to design a process study, the sinking timescales we present are subject to the particle release strategy we have chosen. In the real ocean, clean microplastic would rarely be found in the open ocean, and it could already be weathered, fragmented and biofouled before reaching the subtropical gyres, for example. Including turbulence in the upper layers of the water column would also have an impact on when the particles start sinking and how deep they sink (i.e., probably even sooner and deeper for smaller particles). It is also important to mention that a key result in the Kooi et al. (2017) study is the potential oscillatory behavior of microplastic as a result of biofouling and theoretical defouling below the euphotic layer depth, and this process has yet to be explored further.

To conclude, we have theoretically characterized the effects of biofouling on changing the density of initially buoyant microplastics by estimating the time it takes for the particles to sink, if at all, on a global scale and for a range of plastic sizes and densities. Improving parametrizations of marine microplastic biofouling is crucial to being one step closer to modeling the global ocean plastic budget by understanding biofouling’s role on the fate of the missing 99% of floating marine plastic debris.

## Supporting information

Supporting Information S1

## Data Availability

The NEMO‐MEDUSA‐2.0 ORCA0083‐N06 output for the hydrodynamic data and biogeochemical data used in this study is available from: http://opendap4gws.jasmin.ac.uk/threads/nemo/root/catalog.html. The code to run the simulations is available on Zenodo at: https://doi.org/10.5281/zenodo.4543145.
