# Supplementary material for: Global Modeled Sinking Characteristics of Biofouled Microplastic
Source: J Geophys Res Oceans. 2021 Apr 22;126(4):e2020JC017098. doi: 10.1029/2020JC017098 (PMC8243974; doi:10.1029/2020JC017098)
Supplement: Supplementary file 1 — Supporting Information S1 [file JGRC-126-e2020JC017098-s001.pdf]

Supporting Information for "Global modeled sinking characteristics of biofouled microplastic"

Delphine Lobelle[1], Merel Kooi[2], Albert A. Koelmans[2], Charlotte Laufkötter[3,4],

Cleo E. Jongedijk[5], Christian Kehl[1], Erik van Sebille[1,6]

[1] Institute for Marine and Atmospheric Research, Utrecht University, Utrecht, Netherlands [2] Aquatic Ecology and Water Quality Management Group, Department of Environmental Sciences, Wageningen University, Netherlands [3] Climate and Environmental Physics, Physics Institute, University of Bern, 3012 Bern, Switzerland [4] Oeschger Centre for Climate Change Research, University of Bern, 3012 Bern, Switzerland [5] Department of Civil and Environmental Engineering, Imperial College London, London, United Kingdom [6] Centre for Complex Systems Studies, Utrecht University, Utrecht, Netherlands

Contents of this file

1. Text S1 to S2
2. Figures S1 to S5

S1. Determining the 'most typical year'

The MEDUSA output is available as 5-day averages from 2000 to 2009. Since seasonality is one of the factors that could possibly affect biofouled microplastic sinking, we run four 90-day

Corresponding author: D. Lobelle, Institute for Marine and Atmospheric Research, Utrecht University, Utrecht, Netherlands. (d.m.a.lobelle@uu.nl)

simulations for each of the 10 years over December-January-February (DJF), March-April-May (MAM), June-July-August (JJA) and September-October-November (SON). The particles are released on the first day of the first month of each season. To reduce computationally expensive simulation time, we determine the most typical year using the following steps. Firstly, for each of the 10 years the average sinking timescale (T_s) of the four seasons at each 2° x 2° grid release location is estimated. The 10-year average is then computed at each grid point and this value is subtracted from each year's (4-month-average) values to estimate the anomalies. The absolute sum of all the grid points within each of the global anomaly maps for each year produce 1 value per year. These steps are repeated for four different sizes (10 mm, 0.1 mm, 10 μm, 0.1 μm) while maintaining the initial density of 920 kg m^{-3}. The four normalised single value anomalies per year for each size are summed and the lowest value is chosen as the 'most typical year'; resulting in 2004 being chosen. It is worth mentioning that the global distributions of T_s of all 10 years produce very similar results qualitatively (not shown), hence the process above is carried out simply for robustness.

S2. Additional Kooi model equations

The following equations are extracted from the Kooi et al. (2017) model and produce terms required for eq. (1) and (2) in the main text. As already mentioned, all Kooi model equations related to creating theoretical profiles of temperature, salinity, light or algal concentrations are not included here since we use the output from MEDUSA for these hydrodynamic and biological properties.

Viscosity (kg m^{-1} s^{-1}) can be derived empirically from temperature and salinity profiles following Sharqawy et al. (2010). Dynamic viscosity of water is first calculated, μ_w, followed by

the dynamic viscosity of seawater, μ_{sw}:

$$\mu_w = 4.2844 \times 10^{-5} + \frac{1}{0.156(T + 64.993)^2 - 91.296} \tag{S1}$$

$$\mu_{sw} = \mu_w(1 + AS_z + BS_z^2), \tag{S2}$$

where:

$$A = 1.541 + 1.998 \times 10^{-2}T - 9.52 \times 10^{-5}T^2, \tag{S3}$$

$$B = 7.974 - 7.561 \times 10^{-2}T + 4.724 \times 10^{-4}T^2. \tag{S4}$$

The kinematic viscosity (m^2s^{-1}), υ_{sw}, can then be calculated:

$$\upsilon_{sw} = \frac{\mu_{sw}}{\rho_{sw}}. \tag{S5}$$

For simplicity, the same 1-D kinematic viscosity profile as Kooi et al. (2017) has been used for every gridpoint in our global simulations. This is justified by the fact that kinematic viscosity is dependent on temperature, and for temperatures between 0 and 30 °C, the order of magnitude for υ_{sw} is the same; 1×10^{-6} m^2s^{-1} (Chen et al., 1973). This is an order of magnitude smaller than the smallest vertical velocity throughout the results in this study (not shown), therefore the effects of υ_{sw} on the sinking timescale are minimal.

The volume of the plastic (m^3), v_{pl}, is:

$$v_{pl} = \frac{4}{3}\pi r_{pl}^3, \tag{S6}$$

where r_{pl} is the initial radius of the particle (m) that we choose for each simulation (from the five sizes: 1×10^{-3}, 1×10^{-4}, 1×10^{-5}, 1×10^{-6} and 1×10^{-7} m). The surface area of the particle (m^2), θ_{pl}, is then:

$$\theta_{pl} = 4\pi r_{pl}^2. \tag{S7}$$

The radius of the algae (m), r_a, is calculated assuming both a spherical particle and algal shape:

$$r_A = \frac{3}{4}\left(\frac{v_A}{\pi}\right)^{1/3}, \tag{S8}$$

where v_A is the volume of one algal cell (m^3). The same value as in Kooi et al. (2017) is used; 2×10^{-16} m^3, which is the median from López-Sandoval et al. (2014), with a range of 1.2×10^{-19} to 2.5×10^{-12}. The volume of the biofilm (m^3), v_{bf} can then be estimated:

$$v_{bf} = (v_A A)\theta_{pl}, \tag{S9}$$

where A is the attached algal growth that is dynamically computed in eq. (2) of the main text, and A=0 for t=0. The biofilm thickness (m), t_{bf}, is computed as:

$$t_{bf} = \left(v_{tot}\frac{3}{4\pi}\right)^{1/3} - r_{pl}, \tag{S10}$$

where v_{tot} is the total volume of the plastic particle plus the biofilm (m^3) is simply $v_{pl} + v_{bf}$. Similarly, the total radius of the particle plus biofilm (m), r_{tot} is $r_{pl} + t_{bf}$. This is then used to compute ρ_{tot}, the total density of the plastic plus biofilm (kg m^{-3}):

$$\rho_{tot} = \frac{r_{pl}^3\rho_{pl} + [(r_{tot})^3 - r_{pl}^3]\rho_{bf}}{(r_{tot})^3}, \tag{S11}$$

where ρ_{pl} is the density of the particle (kg m^{-3}) we choose for each simulation (30, 840 or 920 kg m^{-3}). The total surface area of the particle and biofilm, θ_{tot} (m^2) is therefore:

$$\theta_{tot} = 4\pi r_{tot}^2. \tag{S12}$$

The following equations are then required for the diffusivity of the plastic particle and algal cells (m^2s^{-1}), D_{pl} and D_A, respectively:

$$D_{pl} = \frac{k(T + 273.16)}{6\pi\mu_{sw}r_{tot}}, \tag{S13}$$

$$D_A = \frac{k(T + 273.16)}{6\pi\mu_{sw} r_A} \tag{S14}$$

where k is the Boltzmann constant (m^2 kg s^{-2} K^{-1}). The diffusivity equations are required for the encounter kernel rates which are based on the theory of the physical coagulation of marine algal flocs by Jackson (1990). The collision of ambient algae with the particle is a combination of three terms; Brownian motion (β_{brown}), advective shear (β_{shear}) and differential settling (β_{set}) collision frequencies (m^3 s^{-1}):

$$\beta_{brown} = 4\pi(D_{pl} + D_A)(r_{tot} + r_A), \tag{S15}$$

$$\beta_{shear} = 1.3\gamma(r_{tot} + r_A)^3, \tag{S16}$$

$$\beta_{set} = \frac{1}{2}\pi r_{tot}^2 |V_s|. \tag{S17}$$

where γ is the shear rate (2 s^{-1}). The encounter kernel rate, β_A (m^3 s^{-1}), which is included in the first term in eq. (2) in the main text, is therefore $\beta_{brown} + \beta_{shear} + \beta_{set}$.

The next set of equations characterise the buoyancy of the particle which is dependent on its size and density. Here, D_* represents the dimensionless particle diameter:

$$D_* = \frac{(\rho_{tot} - \rho_{sw}) g D_n^3}{\rho_{sw} \upsilon_{sw}^2} \tag{S18}$$

where D_n is the equivalent spherical diameter of the particle and biofilm (m), i.e. $2r_{tot}$. The dimensionless settling velocity, ω_*, can then be calculated as follows:

$$\omega_* = D_*^2 1.71 \times 10^{-4}, \quad \text{for } D_* < 0.05, \tag{S19}$$

$$\begin{aligned} log(\omega_*) \;=\; & -3.76715 + 1.92944\log(D_*) - 0.09815\log(D_*)^2 \\ & -0.00575\log(D_*)^3 + 0.00056\log(D_*)^4, \quad \text{for } 0.05 \le D_* \le 5 \times 10^9 \end{aligned} \tag{S20}$$

$$\omega_* = 1000, \quad \text{for } D_* > 5 \times 10^9. \tag{S21}$$

References

Chen, S. F., Chan, R. C., Read, S. M., & Bromley, L. A. (1973). Viscosity of sea water solutions. *Desalination*, *13*, 37–51.

Jackson, G. A. (1990). A model of the formation of marine algal flocs by physical coagulation processes. *Deep-Sea Research*, *37*(8), 1197–1211.

Kooi, M., Van Nes, E. H., Scheffer, M., & Koelmans, A. A. (2017). Ups and Downs in the Ocean: Effects of Biofouling on Vertical Transport of Microplastics. *Environmental Science and Technology*, *51*(14), 7963–7971. doi: 10.1021/acs.est.6b04702

López-Sandoval, D. C., Rodríguez-Ramos, T., Cermeño, P., Sobrino, C., & Marañón, E. (2014). Photosynthesis and respiration in marine phytoplankton: Relationship with cell size , taxonomic affiliation, and growth phase. *Journal of Experimental Marine Biology and Ecology*, *457*, 151–159. Retrieved from `http://dx.doi.org/10.1016/j.jembe.2014.04.013` doi: 10.1016/j.jembe.2014.04.013

Sharqawy, M. H., Lienhard V, J. H., & Zubair, S. M. (2010). Thermophysical properties of seawater: A review of existing correlations and data. *Desalination and Water Treatment*, *16*(1-3), 354–380. doi: 10.5004/dwt.2010.1079

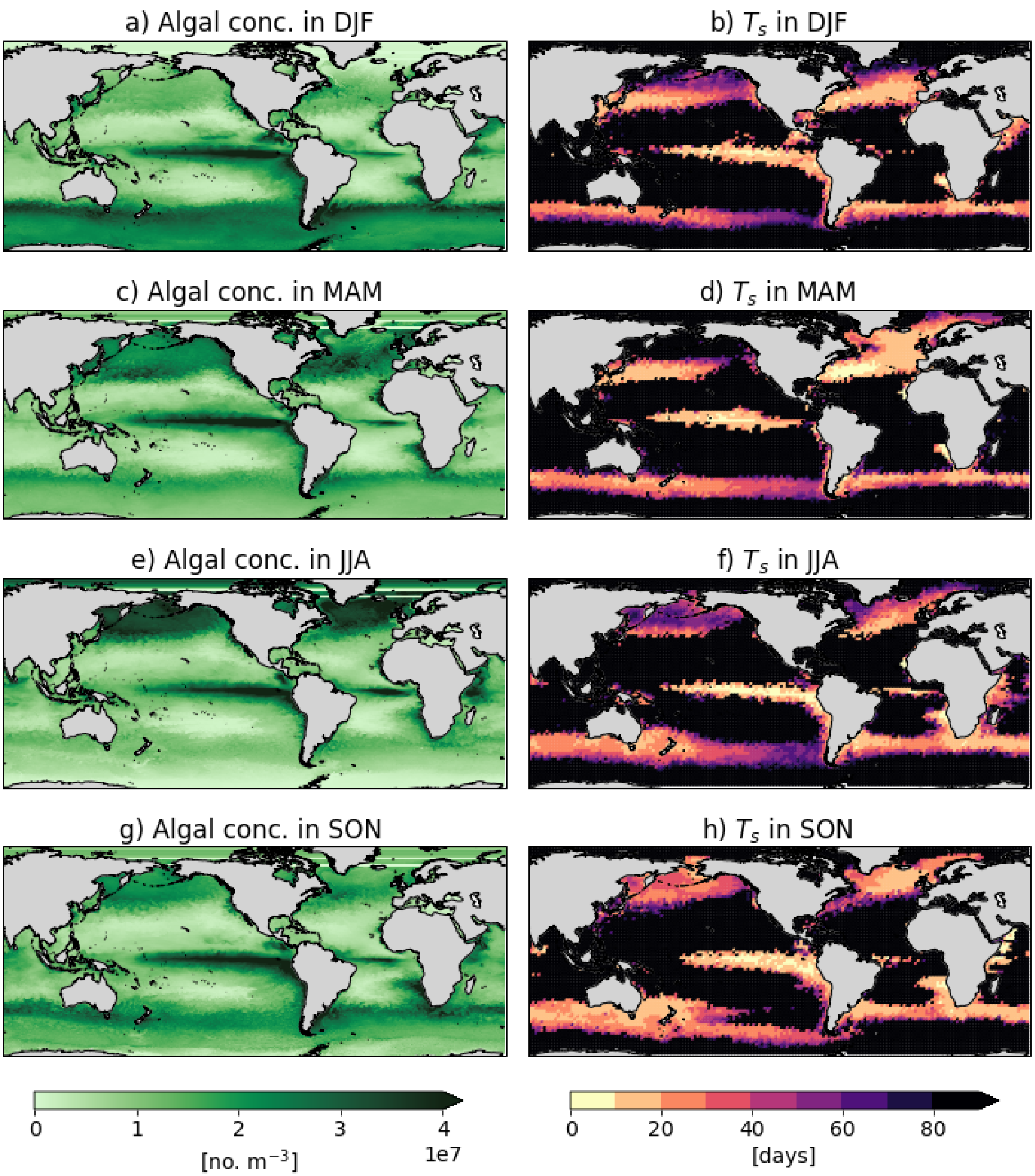

Figure S1. As in Fig. 3 of the main text, but for an initial particle size of 0.1 mm.

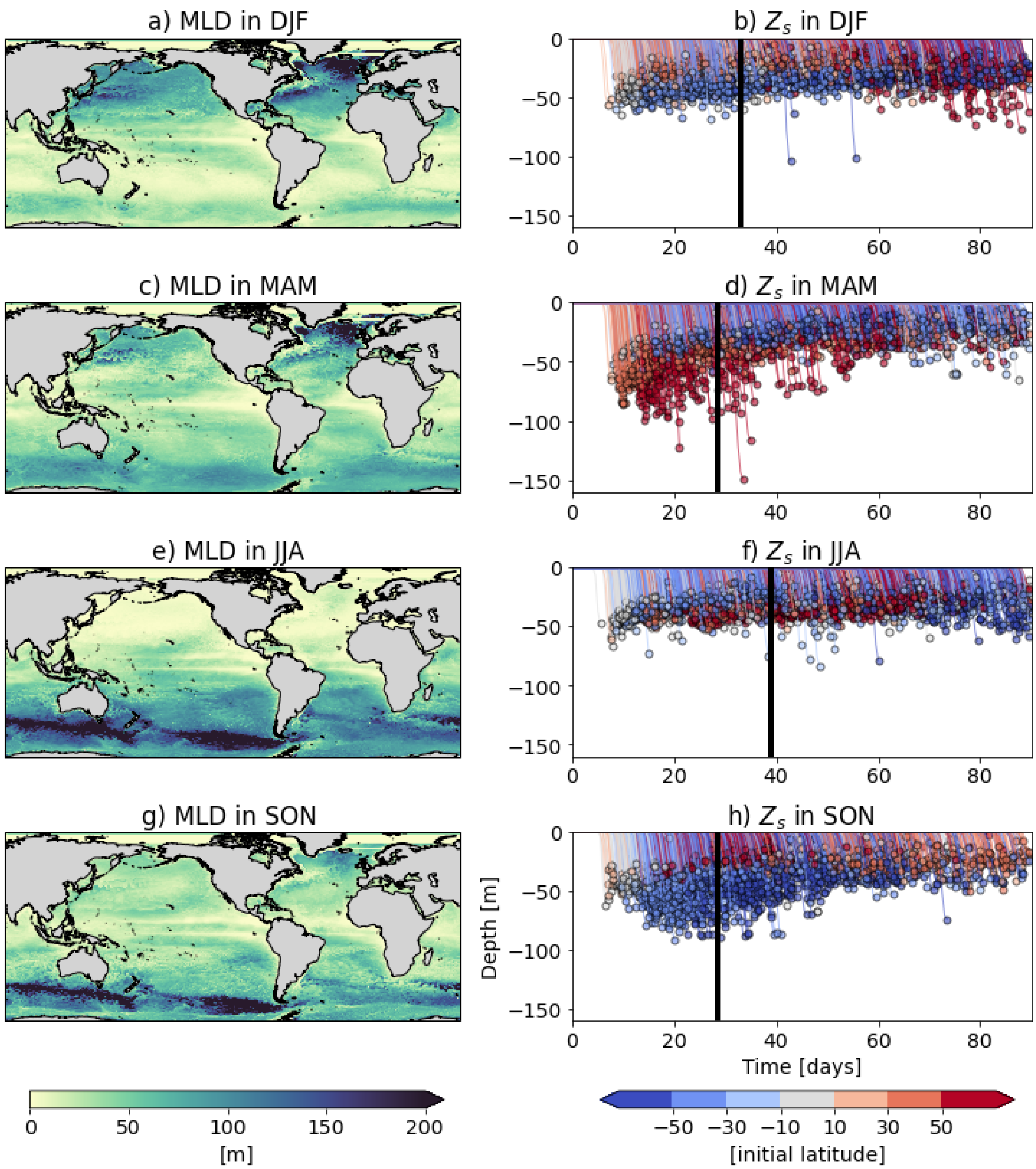

Figure S2. As in Fig. 4 of the main text, but for an initial particle size of 0.1 mm.

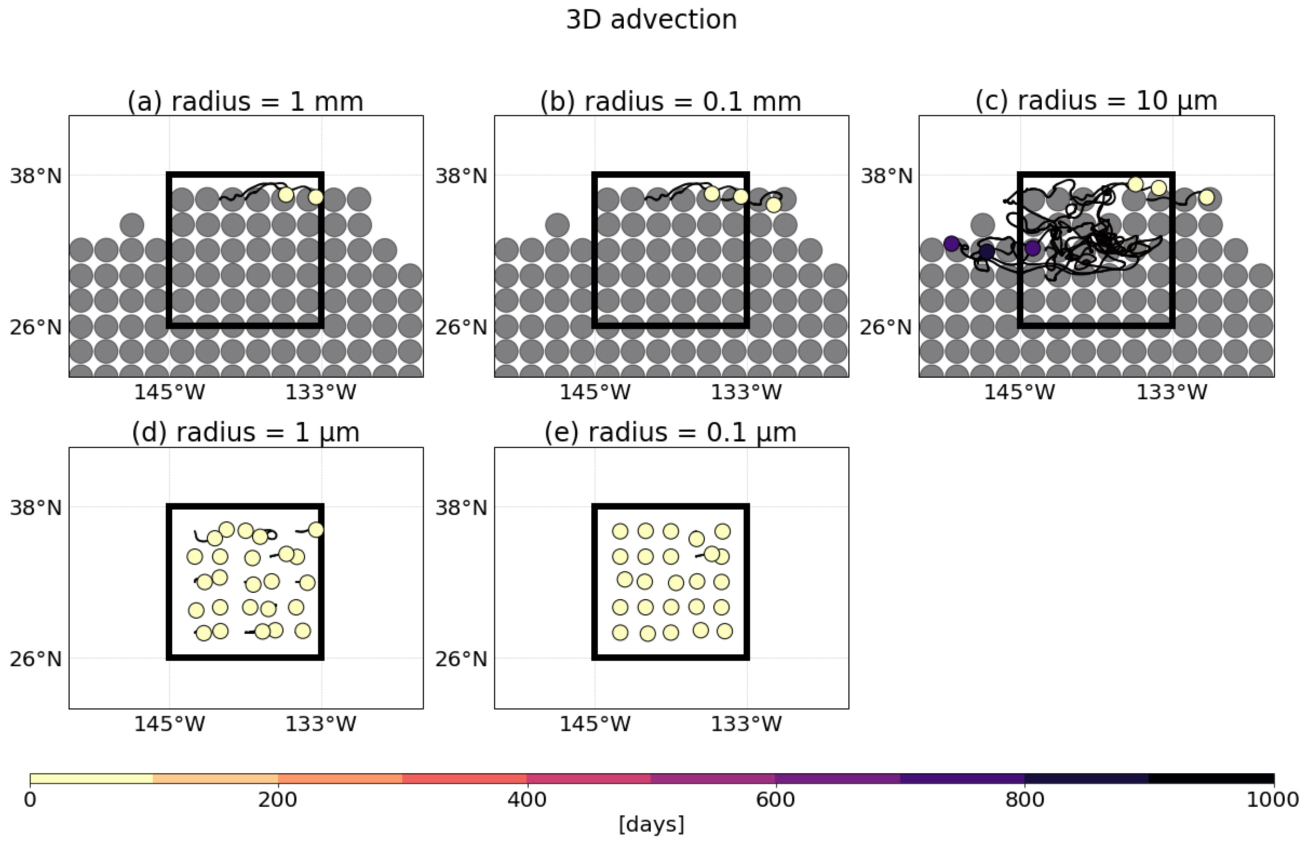

Figure S3. North Pacific Subtropical Gyre 1000–day simulations. The trajectories (black lines) and sinking timescale (T_s in days; colored dot at the end of the black lines) for the 5 particle sizes in the North Pacific subtropical gyre (NPSG). The initial particle density is 920 kg m^{-3} and the initial radii are: (a) 1 mm, (b) 0.1 mm, (c) 10 μm, (d) 1 μm and (e) 0.1 μm. The simulations are run for 1000 days (instead of 90 days in the main text), where the grey dots (in subplots a, b and c) represent the regions where particles do not sink within 90 days in Fig. 1 on the main text. The black box shows the region where the 25 particles are initially released on a 2x2° horizontal resolution. 3D advection has been included to these simulations.

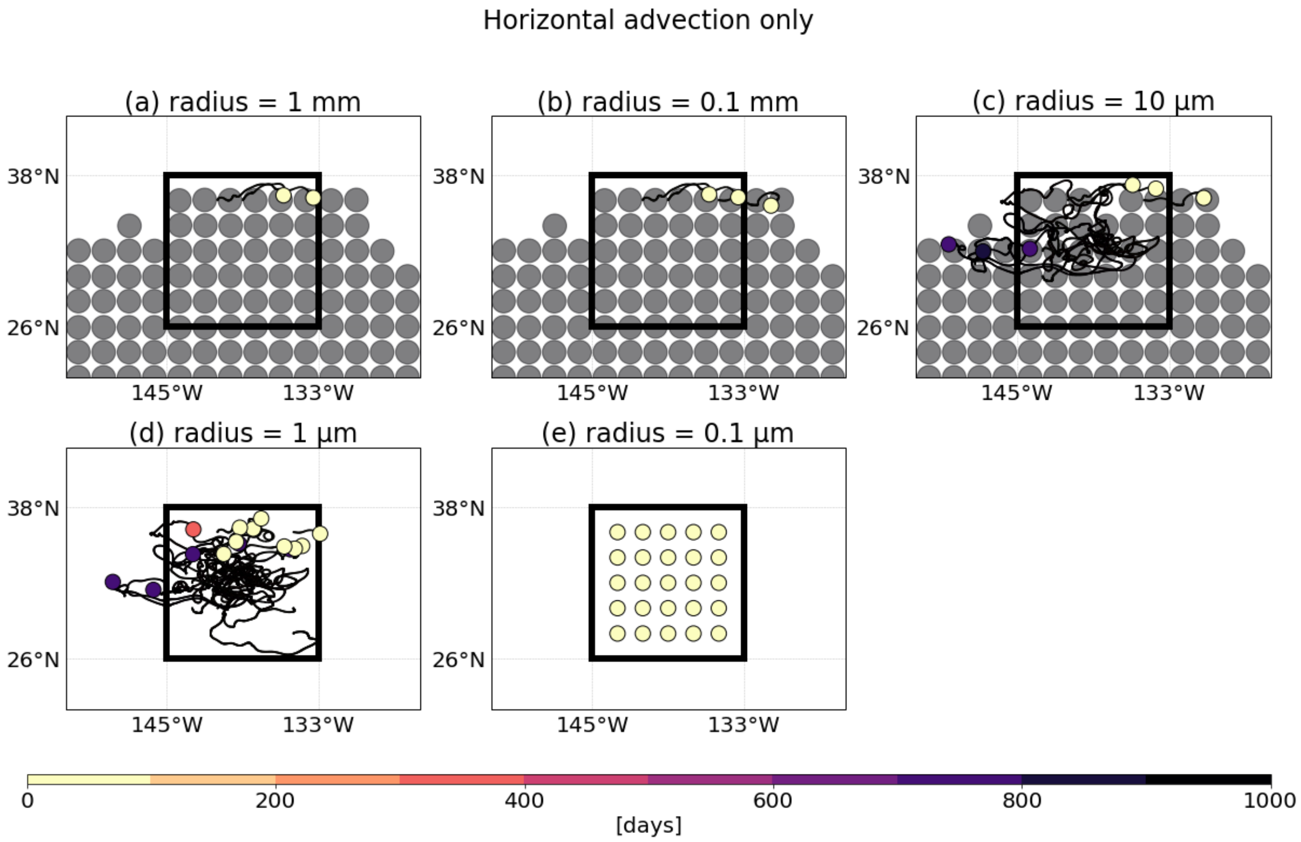

Figure S4. As in Fig. S3 but with horizontal advection only. Here the 1 μm particles in (d) take longer to sink (if at all) due to the lack of vertical advection.

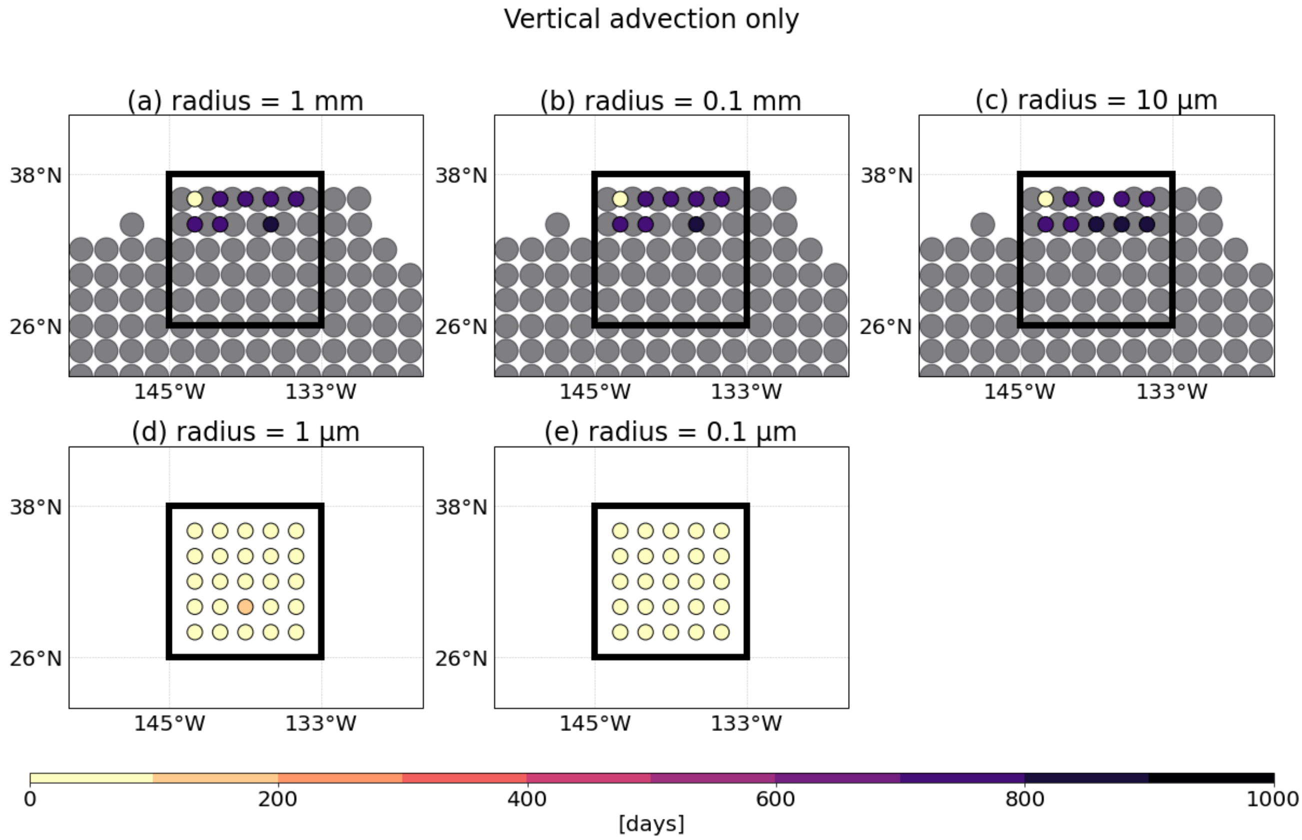

Figure S5. As in Fig. S3 but with vertical advection only. Here the particles are not moved horizontally towards the centre of the convergence zone so larger particles in (a)-(c) are exposed to more algae on the rim of the NPSG (albeit after 700 days, mostly).
